# Maternal Educational Attainment at Birth Promotes Future Self-Rated Health of White but Not Black Youth: A 15-Year Cohort of a National Sample

**DOI:** 10.3390/jcm7050093

**Published:** 2018-05-01

**Authors:** Shervin Assari, Cleopatra Howard Caldwell, Ronald B. Mincy

**Affiliations:** 1 Department of Psychiatry, University of Michigan, Ann Arbor, MI 48109, USA; 2 Center for Research on Ethnicity, Culture and Health, School of Public Health, University of Michigan, Ann Arbor, MI 48109, USA; cleoc@umich.edu; 3 Department of Health Behavior and Health Education, University of Michigan, Ann Arbor, MI 48109, USA; 4 Center for Research on Fathers, Children, and Family Well-Being, New York, NY 10027-5927, USA; rmincysr@gmail.com; 5 Columbia Population Research Center (CPRC), New York, NY 10027-5927, USA; 6 Columbia School of Social Work, New York, NY 10027-5927, USA

**Keywords:** race, ethnicity, self-rated health, social class, education, socioeconomic status, social determinants of health

## Abstract

Background: Socioeconomic status (SES) is essential for maintaining health, and self-rated health (SRH) is not an exception to this rule. This study explored racial differences in the protective effects of maternal educational attainment at birth against poor SRH of the youth 15 years later. Methods: Using data from the Fragile Families and Child Wellbeing Study (FFCWS), this 15-year longitudinal study followed 1934 youths from birth to age 15. This sample was composed of White (*n* = 497, 25.7%), and Black (*n* = 1437, 74.3%) youths. The independent variable was maternal educational attainment at birth. SRH at age 15 was the dependent variable. Family structure was the covariate. Race was the focal moderator. We ran logistic regression models in the pooled sample, as well as stratified models based on race. Results: In the pooled sample, maternal educational attainment and family structure were not predictive of SRH for the youths at age 15. Race interacted with maternal educational attainment, indicating a stronger association between maternal educational attainment at birth on youth SRH for Whites compared to Blacks. In race stratified models, maternal educational attainment at birth was protective against poor SRH for White but not Black youths. Conclusion: White but not Black youths gain less SRH from their maternal educational attainment. Enhancing education attainment may not have identical effects across racial groups. The health status of Blacks may be less responsive to improvements in maternal educational attainment. Policies should go beyond investing in educational attainment by empowering Black families to better use the educational attainment that they gain. Policies and programs should reduce the costs of upward social mobility for minority families.

## 1. Introduction

Despite its simplicity, self-rated health (SRH) is an exceptionally strong predictor of mortality [1,2]. Meta-analyses have found that individuals who report “poor” health are at a twofold higher risk of mortality compared to individuals who report “excellent” health [3]. Poor SRH is found to predict risk of death in more than 80% of the studies [4,5]. This relationship between SRH and mortality persists above and beyond objective indicators of health, including health-related behaviors, chronic disease, function, biomarkers, and access to care [3].

Due to the robust relationship between SRH and mortality, SRH is widely used in epidemiological studies [6,7]. This strong evidence has led some researchers to claim that “an individual’s health status cannot be assessed without SRH” and that this single item captures “an irreplaceable dimension of health status” [4]. While most of the work on SRH is on adults, research has confirmed the validity of SRH in adolescents [8,9,10,11,12]. These studies have shown that SRH is a stable, reliable, and valid health indicator that reflects both the physical and mental health of individuals [2,8,9,10,11,12,13,14].

Social patterning of health (i.e., health closely follows the socioeconomic status (SES) gradient) [15,16,17] is also true for SRH [18]. Although SES indicators have “enduring, consistent, and growing” health effects [19], SES effects differ across various contexts [20]. Although the overall effects of education on population health are shown by several longitudinal studies [21,22,23,24,25], we know less about racial inequalities as related to the protective effects of family SES at birth on youth SRH [26]. The current objective is to try to fill the knowledge gap regarding Black–White differences regarding the effect of family SES at birth on subsequent SRH of youths.

The health effects of SES [27] and the underling mechanisms behind such effects are not universal across populations [28,29,30,31]. This is because marginalized and minority populations face greater societal barriers that hinder them from translating their resources to tangible health outcomes [27,32,33,34,35]. With an assumption that the effects of SES are universal across groups, we know little about racial variations in the health gains that follow SES resources [15,16,17]. What is needed is to expand the existing knowledge from the overall effects of SES [33,36,37,38,39,40] to focusing on the sub-population group variations of such effects [33,38,41,42,43,44,45,46,47,48].

Race also alters how SES indicators impact health [49]. In a recent study, White parents could better use their education to escape poverty compared to Black parents [36]. Also in theory, SES is supposed to enhance a population’s ability to escape and buffer stress, however, in reality, the magnitude of these effects differs across populations. This is because the magnitude of the effects of a particular SES indicator depends on the availability of other resources, which is not equal across demographic groups [50]. As a result, educational attainment does not generate the same change in employment, income, wealth, purchasing power, insurance, and overall life conditions for various sub-populations [51,52,53,54,55].

There is still more to be learned about how Black and White youths differ in the effects of parental SES influences on their SRH [40,52,56,57,58]. A recent 25-year follow-up study showed that high family SES at birth reduces BMI at age 15 among White but not Black youths [59]. Another study documented an increased risk of major depressive disorder (MDD) in high SES male African American youths [60]. Fuller-Rowell et al., have also shown that high SES is associated with greater social costs for Black youths compared to White youths [61], and the health gains of upward social mobility may be smaller for Black than White youths [62]. Similar results have been also shown for adults, as SES has shown smaller protective effects on drinking [51], depressive symptoms [54], suicidality [55], chronic disease [54], and mortality [40,52,57,58] for Blacks compared to Whites. These are all in line with the Minorities’ Diminished Return theory that suggests racial and ethnic minorities gain less health from the same investment than Whites from the same level of SES [63].

To test if the Minorities’ Diminished Return theory [63] also holds true for the effects of maternal education on SRH of youth, this study compared the effect of maternal education on SRH reported by youth at age 15 in Black and White families. In line with the Minorities’ Diminished Return theory [63,64,65,66,67,68,69,70,71,72,73], we expected a smaller effect of baseline SES on SRH at age 15 in Black compared to White families. As family types differ for Whites and Blacks, we aimed to explore whether these variations are due to different family structures or not.

## 2. Methods

### 2.1. Design and Setting

This is a longitudinal study that used data from the first and sixth waves of the Fragile Families and Child Well-being Study (FFCWS), 1998–2016. The FFCWS has enrolled a random sample of urban families from 20 US cities with populations of at least 200,000. Although more detailed information on sampling and data collection of the FFCWS are published elsewhere [74], we briefly summarize the methodology of the study.

### 2.2. Original Sample

FFCWS includes 4655 families (2407 Black, 1354 Hispanics, and 894 Whites). The FFCWS oversampled couples who were non-married [74,75]. As a result, it is not representative to the US general population. Most of the FFCWS participants were non-marital and low SES families.

### 2.3. Analytical Sample

This analysis only included Black and White families with family SES data at baseline (wave 1) and youth SRH 15 years later (wave 6). Family SES was measured at baseline (wave 1). Outcome was youth SRH which was measured when the youth was 15 years old (wave 6). The analytical sample for this study was 1934 youths, composed of 497 Whites (25.7%) and 1437 Blacks (74.3%).

### 2.4. Measures

#### 2.4.1. Main Independent Variables

Family SES indicators at birth were the main independent variables. The present study included two SES indicators, namely, family structure and maternal education, measured at the baseline interview (wave 1). Family structure was a dichotomous variable based on the marital status reported by the mother at wave 1. Maternal education was measured as an ordinal variable: (1) less than high school graduate; (2) completed high school; (3) some college education; and (4) college completed.

#### 2.4.2. Main Dependent Variables

Participating youths were asked to rate their SRH. Response items were excellent, very good, good, fair, and poor. As the literature suggests that dichotomous SRH is the most accurate, SRH was treated as a dichotomous variable. We collapsed the items into two categories (fair/poor; 1 vs. excellent/very good/good; 0), a cut point that is commonly used in the literature [7,76,77,78,79].

### 2.5. Statistical Analysis

Data were analyzed using SPSS 22.0 (IBM Corporation, Armonk, NY, USA). We reported frequency (%) and mean (SD) to describe the sample. We used Pearson’s correlation test to calculate our bivariate correlations in the pooled sample and for each race. We used logistic regression models in the pooled sample and specific to each race. In all models, poor SRH at age 15 was the outcome, and one SES indicator was the independent variable. First, two main effect models were estimated in the pooled sample to test the separate effects of SES indicators (Model 1 and Model 2). Then we ran two additional models (Model 3 and Model 4) that included the race by SES interaction terms. Final models tested the effects of SES indicators in Whites and Blacks, respectively. We also ran models to test the additive effects of marital status and maternal education. Adjusted Odds Ratio (OR) 95% confidence intervals (CI), and *p* values were reported. *P* values of less than 0.05 were considered statistically significant.

### 2.6. Ethics

Princeton University Institutional Review Board approved the FFCWS protocol. The adolescents’ legal guardians and parents signed informed consent. The youths provided assent. Respondents were financially compensated for their participation. 

## 3. Results

### 3.1. Descriptive Statistics

This study followed 1934 youths from birth to age 15. This sample was either Whites (*n* = 497, 25.7%) or Blacks (*n* = 1437, 74.3%).

Table 1 describes the study variables in the pooled sample, as well as by race. Maternal education was higher for Whites compared to Blacks. Most White youths were from families with married parents. Most Black youths were, however, from families with unmarried parents. Finally, SRH at age 15 was worse for Blacks compared to Whites (Table 1).

### 3.2. Bivariate Correlations

Table 2 summarizes the results of three sets of bivariate correlations, which were run in the pooled sample, as well as specific to race. SES indicators were associated with SRH at age 15 in the pooled sample. These associations, however, could only be found for Whites but not Blacks (Table 2).

### 3.3. Separate Effects of Maternal Education and Marital Status

Table 3 summarizes the results of four logistic regression models in the pooled sample and then by race. The first models that did not include any interaction term showed that maternal education was not associated with SRH of the youths at age 15. The next model showed that race interacts with maternal education as related to SRH, suggesting a larger effect for Whites than Blacks. Our stratified regressions showed that the direction of the associations between maternal education and SRH at age 15 was opposite for Whites and Blacks (Table 3).

Table 4 summarizes the results of four logistic regression models in the pooled sample and then by race. The first models that did not include any interaction term, showed that family structures were not associated with SRH of the youths at age 15. The next model that included one interaction term showed that race interacts with family structure as related to SRH, suggesting a larger effect for Whites than Blacks. Our stratified regressions showed that the direction of the association between family structure and SRH at age 15 was opposite for Whites and Blacks (Table 3).

### 3.4. Additive Effects of Maternal Education and Marital Status

Table 5 summarizes the results of four logistic regression models to test the combined effects of SES indicators in the pooled sample and then for Whites and Blacks separately. The first models, which did not include any interaction term, showed that maternal education and family structure were not associated with SRH of the youths at age 15. The next model, which included both interaction terms, showed that race interacts with maternal education but not with the family structure as related to SRH; this suggests a larger effect of maternal education on SRH for Whites than Blacks (Table 5).

## 4. Discussion

This national study revealed Black–White differences in the protective effects of maternal education at birth against poor SRH of the youths at age 15. Blacks did not gain SRH from high maternal education at birth, a finding which is consistent with the Minorities’ Diminished Return theory [59,80,81]. The differential effect of maternal education between Whites and Blacks was not because of racial differences in the family type.

The protective effects of family SES on SRH support the existing literature on the protective effect of SES on developmental and health outcomes [20,25,82,83,84]. High SES is shown to be a predictor of SRH [85,86]. The health effects of SES, however, are non-specific, as they have been documented for a wide range of mental and physical health domains [20,21,22,23,24,25]. These results are also supported by a number of theories and models that collectively suggest family SES at birth would have implications for the offspring’s health decades later. Link and Phelan’s (1995) Fundamental Cause theory conceptualizes low SES as a risk factor for a wide range of physical and mental health outcomes [15,16,17]. On the basis of Bronfenbrenner’s ecological model of human development [64], and the Family Financial Stress Model [67,68,69], poor SES of a family can be considered a strong risk factor for poor outcomes of youths in general. The results were also supported by the Life Course Developmental Approach which considers early SES as a determinant of long-term health effects decades later [70,71,72,73].

The observed Black-White disparities in the protective effects of baseline family SES against poor SRH of youths at age 15 support the Minorities’ Diminished Return theory (i.e., smaller health gains from SES for Blacks over Whites) [27,52,55,84]. In a previous study, the effect of state income inequality on poor health was larger for Whites than Blacks [85]. Similar findings are reported for Black youths [60]. In a nationally representative sample of Black youths, the risk of MDD was higher for African American males from high-income families [60]. Another study showed stronger effects of discrimination on MDD suggesting that high SES may operate as a vulnerability factor for Black families [83]. There are several studies on the lower health and well-being of high SES Black families as well [34,47,48,59,61,62,83].

Previous research has shown that racial groups [37,41,52,53] differ in the health gains associated with SES [51,87,88,89,90,91,92]. It is still unknown why Blacks gain less health returns from their high SES status [28]. Among adults, education has shown smaller effects on drinking patterns [51], BMI, insomnia, physical activity [51], depressive symptoms [54], suicidality [55], and mortality [52] for Blacks than Whites. There are also studies not only showing a lower gain but also an additional risk associated with high SES for Blacks, particularly Black men [54,55].

Multiple explanations are proposed to explain the diminished health returns from SES factors for Blacks. Racism and racial discrimination may have a role. While SES increases expectations and aspirations, Blacks—even those with a high SES—may face systematic barriers that may diminish the health gains associated with their upward social mobility [54]. In a race-aware society, in the presence of discrimination, high aspirations may be detrimental to the health of Black youths, even when they have high SES families. In this view, high SES may become a vulnerability factor for Black families [34,56]. Hudson has shown that discrimination imposed greater psychological costs for high SES Blacks [34]. This finding is shown in Black youths and adults [56,83].

The results should not be interpreted as being indicative of Blacks are not able to turn their SES resources into tangible health outcomes, which would be blaming the victim. These diminished gains from SES are due to a wide range of societal and structural barriers that systematically prevent Black families from benefiting from their available SES resources [93,94,95,96,97,98,99]; a race aware system which fails Black families even when Black families move up the social hierarchy ladder, and pay the costs of upward social mobility [93,94,95,96]. We believe that the way American society is designed and operates is that it constantly maximizes the gain of the majority and Whites and minimizes the gain of minorities, particularly Blacks. Black families with a high education have a lower income, and have a lower purchasing power.

The findings here have implications for policy and research, particularly regarding racial and ethnic disparities in SRH in youths [100,101,102,103,104]. Policies should target structural barriers that systematically hold Blacks back. Policy solutions should go beyond increasing SES by addressing the barriers that high SES minority families face in their lives. Policies are also needed to ensure that minorities gain leverage from their SES resources such as education to gain employment, income, and insurance. While research on the individual level should also explore the role of racial socialization, racial identity, discrimination, social support, culture, and behaviors, there is a particular need to study contextual (neighborhood and school racial composition) as well as policy factors that result in group differences in the health gains from SES. There is also a need to study economic and social policies that can undo the Minorities’ Diminished Returns [56,59,81,83,105,106].

Our study had a few limitations. First, we conceptualized family SES as fixed and did not treat SES as being influenced by time varying covariates. Research should also explore the differential effects of upward and downward social mobility by race. Future research should also test the differential effects of other SES indicators such as employment and wealth. Second, our SES indicators were measured at an individual level. There is a need to study the effects of contextual factors such as neighborhood SES and the variation of racial density across geographic locations. Third, we did not study some of the confounders such as physical health, access to health care, insurance, or health of parents that could confound the link between SES and health of the youths. Forth, this study exclusively focused on race differences; future work might explore how the intersection of race, gender, place, and SES alter determinants of health of the youths. We did not study a comprehensive list of SES indicators. Other factors such as household size, employment, and wealth should be considered in future studies. There is a need to replicate these findings using other cohorts, settings, and outcomes. The sample sizes were asymmetric (497 vs. 1437), which has implications regarding the statistical power for the effects of maternal educational attainment at birth in promoting future SRH in White and Black youths. Despite these limitations, this is one of the very few studies on the differential role of SES on future SRH among adolescents. A major advantage of this study was the national sample, considerable sample size, and 15 years of follow-up data.

## 5. Conclusions

The observed racial differences in the effects of maternal education at birth on youth SRH at age 15 suggests that major racial differences exist in the health gains from SES in the US, with the very same resources resulting in smaller heath gains for Black compared to White youths. Future research should focus on societal barriers and contextual factors in the lives of Black families that prevent them from gaining the maximum health benefits from their SES. Future research should also investigate how the intersections of race, gender, SES, and place differ in the effect of same risk and protective factors on health. Finally, there is a need to find policy solutions that can undo racial disparities in health gains from those with access to same SES resources. These racial disparities are unfair and emphasize a need for public policy and program planning.

## Figures and Tables

**Table 1:** Descriptive characteristics in the pooled sample and by race.

	All (*n* = 1934)		Whites (*n* = 497)		Blacks (*n* = 1437)	
	*n*	*%*	*n*	*%*	*n*	*%*
**Race**						
White	497	25.70	497	100.00	-	-
Black	1437	74.30	-	-	1437	100%
**Gender**						
Male	983	50.83	-	-	-	-
Female	951	49.17	-	-	-	-
Married *^,a^						
No	1464	75.70	199	40.04	1265	88.03
Yes	470	24.30	298	59.96	172	11.97
Education *^,a^						
Less than high school	534	27.63	65	13.08	469	32.66
High school	641	33.16	107	21.53	534	37.19
Some college	499	25.81	139	27.97	360	25.07
College completed or graduate level	259	13.40	186	37.42	73	5.08
**Self-Rated Health (SRH)**						
Excellent–Good	1771	91.57	466	93.76	1305	90.81
Poor/Fair	163	8.43	31	6.24	132	9.19
	Mean	SD	Mean	SD	Mean	SD
Maternal Education *^,b^	2.25	1.00	2.90	1.05	2.03	0.88

* *p* < 0.05 for comparison of Blacks and Whites; ^a^ Pearson’s chi-squared test; ^b^ Independent sample *t-*test.

**Table 2:** Correlations between study variables in the pooled sample and across races.

	1	2	3	4	6
**Pooled sample (*n* = 1934)**					
1 Race (Black)	1	0.01	−0.49 **	−0.38 **	0.05 *
2 Gender (female)		1	−0.01	−0.02	0.09 **
3 Married			1	0.50 **	−0.01
4 Maternal education				1	−0.04 ^#^
6 SRH (dichotomous)					1
**Whites (*n* = 497)**					
2 Female		1	−0.05	−0.04	0.05
3 Married			1	0.55 **	−0.06
4 Maternal education				1	−0.16 **
6 SRH (dichotomous)					1
**Blacks (*n* = 1437)**					
2 Female		1	0.03	−0.01	0.10 **
3 Married			1	0.29 **	0.05 ^#^
4 Maternal education				1	0.01
6 SRH (dichotomous)					1

^#^*p* < 0.1; * *p* < 0.05; ** *p* < 0.01.

**Table 3:** The results of four logistic regression models in the pooled sample and across races on the separate effect of maternal education on subsequent SRH in the youths at age 15.

	OR	95%CI	OR	95%CI	OR	95%CI	OR	95%CI
	All				Whites		Blacks	
Maternal education	0.90	0.75–1.07	0.56 ***	0.40–0.79	0.56 ***	0.40–0.79	1.07	0.87–1.30
Gender (female)	1.38	0.90–2.13	0.29 *	0.11–0.79	1.47	0.70–3.10	2.03 ***	1.40–2.95
Race (Black)	1.91 ***	1.37–2.66	1.90 ***	1.36–2.66	-	-	-	-
Race * Maternal education	-	-	1.91 **	1.28–2.84	-	-	-	-
Intercept	0.06 ***	-	0.21 ***	-	0.25 **	-	0.06 ***	-

Outcome: Self-Rated Health (SRH); Confidence Interval (CI). * *p* < 0.05; ** *p* < 0.01; *** *p* < 0.001.

**Table 4:** The results of four logistic regression models in the pooled sample and across races on the separate effect of family structures on subsequent SRH in the youths at age 15.

	OR	95%CI	OR	95%CI	OR	95%CI	OR	95%CI
	All				Whites		Blacks	
Married	1.13	0.73–1.74	0.62	0.30–1.30	0.62	0.30–1.28	1.50	0.92–2.45
Gender (female)	1.60 *	1.01–2.53	1.11	0.64–1.91	1.50	0.72–3.14	2.01 ***	1.38–2.93
Race (Black)	1.92 ***	1.37–2.67	1.90 ***	1.36–2.65	-	-	-	-
Race * Married	-	-	2.41 *	1.00–5.80	-	-	-	-
Intercept	0.04 ***	-	0.06 ***	-	0.07 ***	-	0.06 ***	-

Outcome: Self-Rated Health (SRH), Confidence Interval (CI). * *p* < 0.05; ** *p* < 0.01; *** *p* < 0.001.

**Table 5:** The results of four logistic regression models in the pooled sample and across races on the combined effects of maternal education and marital status on subsequent SRH in the youths at age 15.

	OR	95%CI	OR	95%CI	OR	95%CI	OR	95%CI
	All				Whites		Blacks	
Maternal education	0.86	0.72–1.05	0.53 ***	0.36–0.78	0.53 ***	0.36–0.78	1.016	0.82–1.25
Married	1.29	0.81–2.06	1.26	0.54–2.90	1.25	0.54–2.88	1.480	0.88–2.49
Gender (female)	1.90 ***	1.36–2.66	1.89 ***	1.35–2.64	1.47	0.70–3.11	2.01 ***	1.38–2.92
Race (Black)	1.51 ^#^	0.95–2.39	0.30 *	0.11–0.81				
Race * Maternal education	-	-	1.91 **	1.23–2.97				
Race * Married	-	-	1.18	0.44–3.16				
Intercept	0.06 ***		0.21 ***		0.25 **		0.062 ***	

Outcome: Self-Rated Health (SRH), Confidence Interval (CI). ^#^ *p* < 0.1; * *p* < 0.05; ** *p* < 0.01; *** *p* < 0.001.
